# Phase Coupled Meta-analysis: sensitive detection of oscillations in cell cycle gene expression, as applied to fission yeast

**DOI:** 10.1186/1471-2164-10-440

**Published:** 2009-09-17

**Authors:** Saumyadipta Pyne, Roee Gutman, Chang Sik Kim, Bruce Futcher

**Affiliations:** 1 Broad Institute of Massachusetts Institute of Technology and Harvard University, Cambridge, MA 02142, USA; 2 Present address: Department of Medical Oncology, Dana-Farber Cancer Institute, Harvard Medical School, Boston, MA 02115, USA; 3 Department of Statistics, Harvard University, Cambridge, MA 02138, USA; 4 Department of Biological Sciences, RCWD, Sookmyung Women's University, Seoul, Republic of Korea; 5 Department of Molecular Genetics and Microbiology, Stony Brook University, Stony Brook, NY 11794, USA

## Abstract

**Background:**

Many genes oscillate in their level of expression through the cell division cycle. Previous studies have identified such genes by applying Fourier analysis to cell cycle time course experiments. Typically, such analyses generate p-values; i.e., an oscillating gene has a small p-value, and the observed oscillation is unlikely due to chance. When multiple time course experiments are integrated, p-values from the individual experiments are combined using classical meta-analysis techniques. However, this approach sacrifices information inherent in the individual experiments, because the hypothesis that a gene is regulated according to the time in the cell cycle makes two independent predictions: first, that an oscillation in expression will be observed; and second, that gene expression will always peak in the same phase of the cell cycle, such as S-phase. Approaches that simply combine p-values ignore the second prediction.

**Results:**

Here, we improve the detection of cell cycle oscillating genes by systematically taking into account the phase of peak gene expression. We design a novel meta-analysis measure based on vector addition: when a gene peaks or troughs in all experiments in the same phase of the cell cycle, the representative vectors add to produce a large final vector. Conversely, when the peaks in different experiments are in various phases of the cycle, vector addition produces a small final vector. We apply the measure to ten genome-wide cell cycle time course experiments from the fission yeast *Schizosaccharomyces pombe*, and detect many new, weakly oscillating genes.

**Conclusion:**

A very large fraction of all genes in *S. pombe*, perhaps one-quarter to one-half, show some cell cycle oscillation, although in many cases these oscillations may be incidental rather than adaptive.

## Background

Cells reproduce and divide using an ordered set of processes. The cell division cycle is usually divided into four phases, called G1 (Gap 1), S (DNA Synthesis), G2 (Gap 2) and M (Mitosis). In late G1 phase, cells commit to a round of cell division, and in other ways prepare for the upcoming duplication; in S phase they replicate their DNA; in G2 they prepare for mitosis, and during mitosis they segregate their chromosomes, form two nuclei around these two sets of chromosomes, and finally the two new cells separate from one another. These ordered processes are extremely complex, involving hundreds if not thousands of proteins. These processes are regulated and assisted by changes in gene transcription: that is, many genes needed for DNA synthesis are transcribed just before S phase; many genes needed for mitosis are transcribed just before M phase, and so on. Genes regulated in this way - i.e., expressed at a particular time in the cell division cycle, with the effect of aiding progress through a particular part of the cell division cycle - are called cell cycle regulated genes.

In principle, there might be two kinds of genes whose expression oscillates as a function of progress through the cell cycle. What we will call adaptive cell cycle regulation refers to regulation that has the effect of aiding progress through a particular part of the cell cycle, for instance the up-regulation of DNA ligase or histone expression during the DNA synthesis phase. One might expect natural selection in favor of such regulation. In addition, however, there might be what we will call "incidental" cell cycle oscillation, where a gene oscillates in expression, not because the oscillation is directly helpful to the cell, but as a consequence of something else. For instance, chromatin typically condenses in preparation for mitosis, and this condensation might interfere with the transcription of some genes. Such genes would oscillate as a function of the cell cycle, but the oscillation would not be adaptive. In view of the fact that the cell division cycle entails massive changes in the conformation of the DNA, it might not be surprising if a large proportion of genes oscillated for incidental reasons. In this work, we will usually refer to changes in gene expression through the cell cycle as oscillations, and reserve the phrase "cell cycle regulation" for adaptive regulation leading to oscillation. That is, some cell cycle oscillations are due to cell cycle regulation, while other oscillations may be incidental.

The development of microarrays allowed changes in gene expression to be monitored for all genes in a genome, and microarray technology has been applied to the problem of identifying cell cycle oscillating genes in various organisms [1-5]. In a typical time course experiment, a population of cells is obtained such that all the cells are "synchronized" in one particular phase of the cell cycle, such as G1 phase. The synchronized population is allowed to grow and progress through the cycle, and samples of cells are taken at regular intervals as the cells pass through one, two, or even three consecutive, synchronous rounds of cell division. Messenger RNAs are extracted from the cells in each sample, and these are analyzed using microarrays so that the pattern of expression over time can be determined for each gene. For a cell cycle oscillating gene, it is expected that gene expression will peak and trough once per cell cycle, and the oscillation can be modeled using Fourier analysis [4]. There are many variations of this approach, and these have been systematically compared by de Lichtenberg et al [6]. Using methods of this general kind, even the earliest studies found that a significant fraction of all genes oscillate through the cycle. For instance, Spellman et al. found 800 cell cycle oscillating genes in the yeast *S. cerevisiae*, which is more than 10% of the total genes (about 6,000). Because the number of cell cycle oscillating genes found is statistically limited by the amount of data and by the noise in the experiments, it is quite possible that the true number of cell cycle oscillating genes could be larger.

In recent years at least ten microarray time course experiments from three labs have studied cell cycle expression in the fission yeast *Schizosaccharomyces pombe *(Rustici et al. [7], Peng et al. [8], Oliva et al. [9]). This has made *S. pombe *currently the organism with the largest amount of cell cycle transcriptome data. These data sets can be combined by various techniques of statistical meta-analysis. The traditional method of combining cell cycle data sets has been to calculate a p-value (generally based on Fourier analysis and permutation testing) for oscillation of each gene in each individual cell cycle time course, and then combine these p-values with a traditional statistical method such as Fisher's inverse chi square method or Stouffer's sum of *Z*'s method [10]. Marguerat et al. [11] analyzed all ten data sets, and by combining p-values (for periodicity of oscillation and also for expression regulation, see below), they found about 500 cell cycle oscillating genes (out of about 5000 total genes).

However, we argue here that simply combining p-values is an inefficient approach, which ignores a critical source of information in the available data. Consider a putative cell cycle regulated gene, perhaps involved in S-phase. There are two independent hypotheses about such a gene. First, it will peak and trough once per cell cycle, and thus it will generate a relatively large Fourier sum with a small p-value. This is the hypothesis tested by the standard methods of analysis, including the study by Marguerat et al. But in addition, it can also be predicted that in multiple independent time course experiments, the peak of expression will always be at a similar time in the cell cycle. For instance, a cell-cycle regulated gene involved in DNA synthesis will typically reach peak levels of expression just before S-phase (DNA synthesis), while a cell cycle regulated gene involved in mitosis will typically reach peak levels of expression just before M phase. The second hypothesis - that there is a particular, consistent, time of peak expression for a cell cycle oscillating gene - is not tested by current methods; we show that, in conjunction with the earlier hypothesis, it can allow for a more powerful test of cell cycle oscillation.

There are major intuitive advantages of testing the peak phase consistency hypothesis. Microarray noise often affects gene expression readout; yet it is less likely to hide the peak signals in a time course. Moreover, if a gene peaks at the same phase in multiple independent experiments, it is highly unlikely for all the consistent peaks to be affected simultaneously by random noise.

To emphasize the phase consistency hypothesis in a different way, let us consider two different genes, and 10 independent cell cycle time course experiments. Suppose that the times of peak expression in the cell cycle are calculated for each gene in each time course. Let these times be converted to phase angle values in the circular range 0° to 360° (equivalently, the interval of [0, 2π) radians), which corresponds to one complete cycle, and assign 0° to the end of mitosis. For the first gene, let the times of peak expression in the 10 time course experiments occur at 10 different cell cycle times, scattered around the cell cycle. For the second gene, let the times of peak expression in the 10 time courses all occur in S-phase, at about 90° in this example. Then for these two genes, based on the variances of their phase angles, one could conclude that the second but not the first gene oscillates, even without knowing p-values.

**Figure 1:**
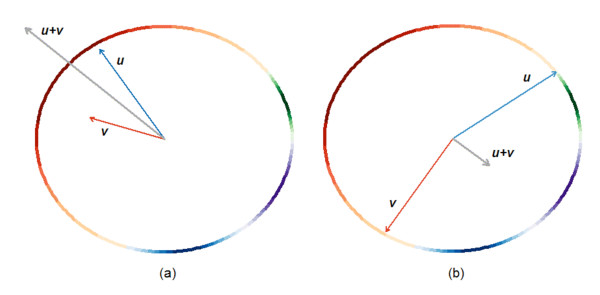
**Vector sum based on oscillation and phase**. For a gene, each time course is represented by a vector such that the Periodicity and Expression scores determine the magnitude of the vector, and the cell cycle phase of peak expression gives the direction of the vector. Plot (a) shows two hypothetical time courses with similar phases of peak expression (both peaking in G2 phase). In (a), the sum of the two vectors ***u+v ***yields a vector (plotted in grey) with a greater magnitude than either of the component vectors. Plot (b) shows two time courses with dissimilar phases of peak expression, one peaking at the beginning of M phase, and the other peaking just after S-phase. The sum of these two vectors ***u+v ***yields a vector (in grey) with a lower magnitude than either of the component vectors. The colored circumference refers to the circular distribution of cell cycle phases in *S. pombe *(red-G2, green-M, purple-G1, blue-S) in both plots.

Here, we propose a new method for the analysis of cell cycle oscillating genes. This new method takes into account both kinds of available information. That is, it uses the standard measures for determining the significance of a gene's oscillation, but in addition, it uses the reproducibility of the cell cycle phase of its peak expression. First, the time course of each gene in each experiment is represented as a vector whose magnitude captures the oscillation information and whose direction captures the peak phase information. Then we combine these two independent sources of information using vector addition; if a gene peaks or troughs in all experiments in roughly the same phase of the cell cycle (we call this property "phase consistency"), then the vectors add to produce a large final vector. Conversely, when the peaks of different experiments are in different phases of the cycle, vector addition produces a small final vector. Fig. 1 shows an example of this kind of analysis over two time courses. The gene shown in Fig. 1a has two time courses in which the phase of peak expression is similar between the time courses, and the resultant vector has large magnitude. The gene shown in Fig. 1b has two time courses in which the phase of peak expression is not similar, and the resultant vector has small magnitude.

To demonstrate the power of this approach, we re-consider the 10 cell cycle time course experiments of S. pombe. We use the same data as Marguerat et al., and we use roughly (though not exactly) the same approach to determine p-values for oscillations in the individual time courses. However, with this new meta-analysis, we find roughly 2,000 cell cycle oscillating genes in *S. pombe*, in contrast to the 500 found by more traditional methods (e.g. Marguerat et al. [11]), a difference we attribute largely to the increase in power due to consideration of peak phase consistency in addition to the p-value of the oscillation. We call this new method "Phase Coupled Meta-analysis", or PCM.

## Methods

Marguerat et al. [11] have already performed a meta-analysis of the *S. pombe *cell cycle data using methods that take into consideration the strength of the oscillations in gene expression. Because we particularly want to highlight the effect of adding phase information, in general we have used the same data and followed the same methods as Marguerat et al. for calculating the strength of oscillations.

### Experiments and data

The ten microarray cell cycle experiments used in the present study were from Rustici et al. 2004; Peng et al. 2005; and Oliva et al. 2005 [7-9], and were the same experiments used by Marguerat et al. [11]. Most of these time courses covered more than one round of cell division. In their meta-analysis, Marguerat et al. used a small set of experimentally validated cell cycle regulated genes to align the cell cycle stages of all ten experiments. They expressed the time of peak expression for each gene in each time course as a percentage of the elapsed cycle, after aligning the various time courses. Using their estimated cell cycle period for each experiment, we transformed these peak times of expression into angular values in the circular range [0, 2π) radians. This produced our input matrix of 4990 × 10 angular data points where the (*i*, *j*)^th ^entry represented the cell cycle phase of peak expression for gene *i *in experiment *j*. In addition, genome-wide data from ten transcription factor knockout and over-expression experiments were used for clustering (see below), which are described elsewhere [9].

### Periodicity and Expression scores

Following de Lichtenberg et al. and Marguerat et al. [6,11], we evaluated the Periodicity score (*P*_*gj*_) of each gene *g*'s time course in the *j*^th ^experiment (*j *= 1...10) using a Fourier sum, and its Expression score (*E*_*gj*_) with standard deviation. For assessment of global significance of the periodicity and expression scores, we used permutation testing. Since by shuffling only along the time axis, the standard deviation of a time course (and hence its expression score) does not change, we adopted a different but related strategy. We generated a joint null population of 10^5 ^permuted simulated time courses by shuffling the original time courses in each experiment both along the gene and time axes, thus destroying any signals from biological regulation. Then with respect to these 10^5 ^randomly-generated "genes" we computed the percentile scores *qP*_*gj *_and *qE*_*gj *_for every real gene *g*. That is, for every real gene, we compared its Periodicity score and Expression score to the scores of the joint null population. The scores for the real genes were then ranked. The percentiles *qP*_*gj *_and *qE*_*gj *_were computed as  (*P*_*gi*_) and  (*E*_*gi*_) respectively using the cumulative distribution functions ( and ) of the null population's periodicity and expression scores for experiment *j*. The cumulative distribution functions were computed with the R function *ecdf*. As percentiles, *qP*_*gj *_and *qE*_*gj *_assumed values between 0 that represented minimal statistical significance and 1 that represented the gene that is most strongly periodic or expressed. Note that the percentile scores allowed a direct genome-wide ranking mechanism with built-in nonparametric statistical significance owing to the background provided by the joint null distribution.

### Phase Coupled Meta-analysis score

Since a cell cycle oscillating gene with a true phase of peak expression is likely to peak consistently in multiple independent experiments, the phase consistency should cause the oscillatory signals to "add up" in a phase coupled meta-analysis measure. Thus we define our Phase-Coupled Meta-analysis (PCM) score for a gene *g *as the magnitude of a sum of *L*_*g *_(≥ 5) vectors, *PCM*(*g*) = , where the *j*^th ^vector ⟨*M*_*gi*_, θ_*gi*_⟩ of *g *has direction θ_*gj *_given by the phase angle of *g *in experiment *j *and magnitude *M*_*gj *_defined as *M*_*gi *_= exp (*qP*_*gi*_) × exp(*qE*_*gi*_) based on the percentile scores described above. The exponential spreading out of their percentile scores allowed the genes to be ranked more distinctively, in particular those with weak expression. For instance, the magnitude a gene that is both strongly periodic and strongly expressed is higher than that of a gene with strong periodicity but weak expression by an exponential factor. Moreover, the exponential function's lower bound of 1 (i.e. for the 0^th ^percentile), instead of 0, allowed even the weak expression profiles to be included in the PCM score; only if a gene *g *is not present in some experiment *j*, the corresponding magnitude *M*_*gj *_is zero. Finally all genes were ranked in the decreasing order of PCM scores, which we refer to as the PCM rank list (see Additional file 1).

### Circular statistics

Circular variance, test of circular uniformity and sampling from von Mises distribution were computed with the R package CircStats. P-values based on the test of circular uniformity were adjusted for multiple hypotheses testing by Benjamini-Hochberg procedure [12]. The median phase angle [13] of every gene was computed as a solution to the following optimization: , where *L*_*g *_is the number of experiments in which the gene *g *is present. Genes that were absent in more than five experiments (i.e. *L*_*g *_< 5) were excluded from the PCM analysis (marked as 'NA' in the PCM rank list).

To evaluate the extent of phase consistency among *S. pombe *oscillations, we compared every gene's PCM score with its simulated version (PCM*) with known phase variance. The simulated score was computed for each gene *g *by using the original oscillation information {*M*_*gi*_: *j *= 1,... *L*_*g*_} from every experiment but randomizing the original phase information {θ_*gi*_: *j *= 1,... *L*_*g*_}: PCM*(*g*) = , by taking the median over fifty random samplings of phases  from the von Mises distribution *VM*(*μ*_*g*_, *k *= 1) with mean *μ*_*g *_specified by circular mean of the observed phases {θ_*gi*_: *j *= 1,... *L*_*g*_} and unit circular variance (1/*κ *= 1). Then we computed the difference *PCM*(*g*)-PCM*(*g*) as a measure of deviation of the observed phase variance of a gene *g *from a corresponding distribution with fixed circular variance.

### Clustering

For clustering, we used the following two-step refinement strategy --

***Step 1***: we clustered the top 2,000 genes from the PCM rank list using data from the ten transcription factor knockout and over-expression experiments to identify genes with similar regulatory signatures. We used the Partitioning Around Medoids (PAM [14]) algorithm, a version of k-means clustering that is robust against outliers. The optimal number of clusters in this step (found to be 8) was determined by maximizing the Average Silhouette Width [15].

***Step 2***: we extended and used the recently developed Phase Synchronization Clustering (PSC [16]) algorithm on the ten time course experiments to classify the clusters from step 1 into groups of genes having specific peak phase expression. In particular, we focused on a cluster of 551 genes composed of two PAM clusters from step 1 having similar regulatory signatures and containing 43 out of 44 genes from the original ribosomal biogenesis cluster in Oliva et al. The extended PSC algorithm produced a phase-specific cluster of 103 genes, which peaked in mid G2 phase of the cell cycle, as an enhanced version of the original ribosomal biogenesis cluster of Oliva et al. (consequently we named it *C*_*ribo*_). The genes in *C*_*ribo *_are indicated in the PCM rank list (Additional file 1).

In step 2, we extended the original multivariate PSC algorithm of Kim et al. [16] in two ways to construct the enhanced ribosomal cluster *C*_*ribo *_with greater statistical power and precision. In the original PSC algorithm, the strength of phase similarity (or "synchronization") between the time courses of two genes *g *and *g' *was measured by the mean phase coherence ρ_*gg*' _(see [16] for details), which assumed values between 0 for no phase synchronization, and 1 for perfect synchronization. Then the expression ρ_*gg*' _was extended to compute phase synchronization ρ_*gC *_between a gene *g *and a cluster *C *by substituting the phase of *g' *with the weighted mean phase of *C*, denoted by Φ_*C*_, such that the weights measured how closely each gene *g *in cluster *C *followed the common phase Φ_*C*_. For details, see [16] and [17].

In the present study, we incorporated the idea of *consensus *about a gene's phases in multiple experiments by computing the mean phase coherence [16] ρ_*gC *_for a given gene-cluster pair over five or more experiments. Another extension to the PSC algorithm was the idea of *seeding *the mean phase Φ_*Cribo *_of the target cluster *C*_*ribo *_with the phases of the original ribosomal biogenesis genes with the aim of identifying additional and possibly weakly oscillating genes which synchronized with the original ribosomal cluster in five or more experiments. The above-mentioned 551 genes (based on the clusters in Step 1), with regulatory signatures similar to the original 43 ribosomal genes, were further clustered with the extended PSC algorithm (with the cutoff parameter set to 0.6; see [16] for details on this parameter). Eight outlier genes with high phase variance were removed, resulting in a cluster of 103 genes (called *C*_*ribo*_).

### Motif analysis and orthology analysis

The enhanced cluster *C*_*ribo *_allowed us to investigate the regulatory mechanism underlying it. No motif was reported for the ribosomal cluster in [9]. The powerful motif search tool BioProspector [18] was applied to the full upstream intergenic regions of the 103 genes in *C*_*ribo *_as foreground DNA sequences, and all intergenic regions in *S. pombe *were used as the background DNA sequences. To identify phylogenetically conserved binding sites, the upstream intergenic sequences of the *C*_*ribo *_orthologs in another fission yeast species *Schizosaccharomyces japonicus *were obtained from the *Schizosaccharomyces *group Database at the Broad Institute of MIT and Harvard University. The motif visualization program MotifViz [19] was used to detect the transcription factor binding sites with motif match p-value cutoff 0.01 and with all intergenic sequences of *S. pombe *and *S. japonicus *as background sequences for searching with the respective species. The TRANSFAC database [20] was searched for similar transcription factor binding sites in other species. The orthologs were identified by reciprocal best hits among species with the program Inparanoid [21]. For *S. pombe *orthologs of cell cycle oscillating genes in other species (human, *S. cerevisiae *and *Arabidopsis*) we integrated data from multiple sources (the databases YOGY [22] and Cyclebase.org [23]; also unpublished orthology data from Chris Penkett).

## Results

**Figure 2:**
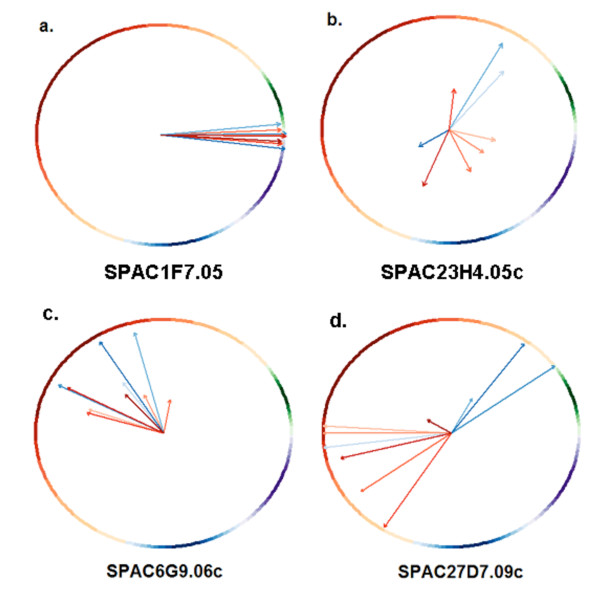
**Four examples genes**. A variety of cases are observed: in (a), SPAC1F7.05 (*cdc22*, ribonucleotide reductase) has both high Periodicity and Expression scores, and also high phase consistency. It is the highest-ranked gene by PCM (rank 1). In (b), SPAC23H4.05c has low Periodicity and Expression scores, and also low phase consistency, leading to a very low PCM rank (4000). In (c), SPAC6G9.06c (*pcp1*) has mediocre Periodicity and Expression scores, but relatively high phase consistency (PCM rank of 259), and finally (d), SPAC27D7.09c (encoding a but1 family protein) has mostly high Periodicity and Expression scores, but relatively low phase consistency (PCM rank of 1464). In some cases, vectors were intentionally offset by small amounts to avoid overlapping. The colored circumference refers to the circular distribution of cell cycle phases in *S. pombe *(red-G2, green-M, purple-G1, blue-S), while the vectors in colder (bluish) and warmer (redish) hues represent cdc25 and elutriation experiments respectively.

**Figure 3:**
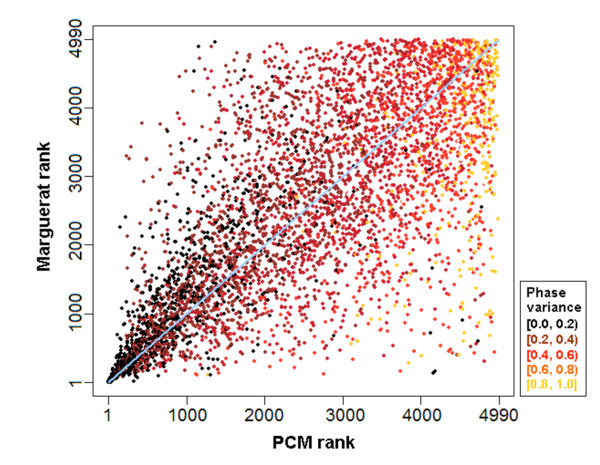
**Specificity and sensitivity of PCM ranks**. Each gene is plotted as a 2-dimensional point where the *x*-coordinate is its PCM rank and the *y*-coordinate is its Marguerat et al. rank. Genes with higher phase consistency are marked with darker points. Clearly there are many more darker points above the diagonal (*y *= *x*) line, suggesting that genes with consistent phases across experiments get higher ranks (i.e. ranked closer to the top) by PCM than Marguerat et al. The sparse upper left quadrant of the plot shows that if genes did not receive a high rank by Marguerat et al., due to their poor Periodicity and Expression scores, then they did not get high a PCM rank either. However, many genes in the lower right quadrant received a high score from Marguerat et al. on the basis of good Periodicity and expression scores, but a low PCM score on the basis of poor phase consistency.

**Figure 4:**
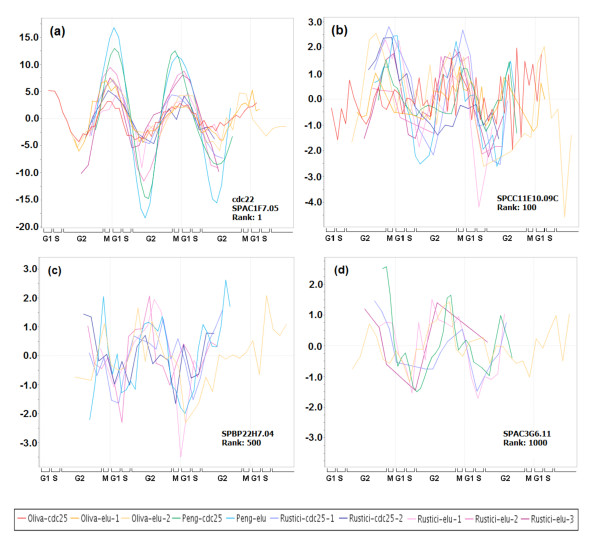
**Variation in peak phase consistency captured by PCM ranking**. Time courses of genes with different PCM ranks are shown: (a) rank 1; (b) rank 100; (c) rank 500; and (d) rank 1000. High peak phase consistency among the ten independent time courses can be seen for the high PCM ranked genes in plots (a) and (b), while in the lower PCM ranked genes peak phase consistency is less, which can be observed among fewer time courses ((c) and (d)). (The plots in this figure were created with the help of Cyclebase.org due to Gauthier et al.)

For each gene and time course, we coupled cell cycle phase information with the standard measures of periodicity and expression to derive a new Phase-Coupled Meta-analysis measure (PCM score). The cell cycle oscillation of a gene *g *in a particular experiment *j *is represented by a vector ⟨*M*_*gi*_, θ_*gi*_⟩ with magnitude *M*_*gj *_directly proportional to the gene's periodicity and expression scores, and direction θ_*gj *_given by the gene's peak phase angle within the cell cycle (Fig. 1). If *g *shows consistency in peak phase over many experiments, then its vector sum (which is also a vector) will be larger in magnitude than any component vector (Fig. 1). The PCM score for a gene is the magnitude of the vector sum over all the experiments (see Methods); it was computed for every gene that was present in at least five out of ten experiments. The genes were then ranked by their PCM scores, and these ranks were compared to the ranks of the same genes obtained by Margureat et al. [11]. The ranked list is available as Additional file 1. Fig. 2 shows results for four example genes. These demonstrate all four possible combinations of large or small vector magnitude, and high or low phase consistency. Fig. 3 shows the correlation between PCM ranks and Marguerat ranks. It is of course expected that they are well correlated, since the Marguerat Periodicity and Expression scores are used to help calculate the PCM score. Genes with relatively low magnitude vectors, but high phase consistency (Fig. 2c) have much better PCM ranks than Marguerat ranks (shown by dark points high above the diagonal line in Fig. 3). It is particularly genes of this kind that are found as cell cycle oscillating genes by our analysis, but missed by methods that focus only on the strength of the oscillation in expression. Genes with relatively large magnitude vectors, but poor phase consistency (Fig. 2d), have much worse PCM ranks than Marguerat ranks, and are found in the lower right quadrant of Fig. 3. These genes are not considered cell cycle oscillating by PCM analysis, but might be so considered by other methods. Fig. 4 shows the time course data for four example genes. The examples chosen had PCM ranks of 1 (i.e., the most strongly regulated gene, *cdc22*, also see Fig. 2a), 100, 500, and 1000. By rank 1000, oscillations (at least when presented in this format) are barely visible to the eye, but nevertheless are statistically detectable.

### Number of Genes showing Cell Cycle Oscillation

In principle, one could imagine that there might be genes regulated by the cell cycle, which might have strong or weak oscillations, and in addition there might be other genes that do not oscillate. Thus there might be a biphasic distribution of oscillation. However, previous genome-wide cell cycle transcriptional studies have not supported this [4,8,9]. Instead, these studies show a small number of genes with strong oscillation, a larger number of genes with slightly weaker oscillation, an even larger number of genes with still weaker oscillation, and so on, until the statistical evidence for cell cycle oscillation reaches the level of noise in the underlying experiments (e.g., Fig. 2 of ref [9]). That is, it appears there is just one population of genes, and it varies in a continuous manner from strongly oscillating to very weakly oscillating or not oscillating at all. Various authors have drawn a more-or-less arbitrary cut-off at some point on the distribution to define a set of "cell cycle regulated" genes. In *S. pombe*, this arbitrary number of genes thought to be cell cycle regulated was 407 (Rustici), 747 (Peng), 750 (Oliva) or 500 (Margureat).

**Figure 5:**
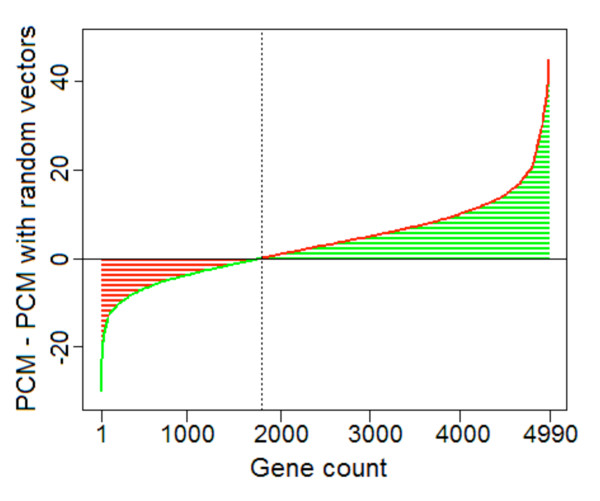
**Difference of PCM scores with real and random phases**. For each gene, the PCM score was computed as sum of vectors (see Methods). Then for the same gene, a random-phase PCM score was computed as sum of vectors with the original magnitudes but randomly-chosen phase angles. For every gene, the difference between its original PCM score and its random-phase PCM score was then computed, and plotted in increasing order from left to right. To the right of the dotted line are 3,200 genes where the difference is positive; i.e., for these genes, the cell cycle phases of peak expression in the time courses are less variable than the randomly distributed phases.

**Figure 6:**
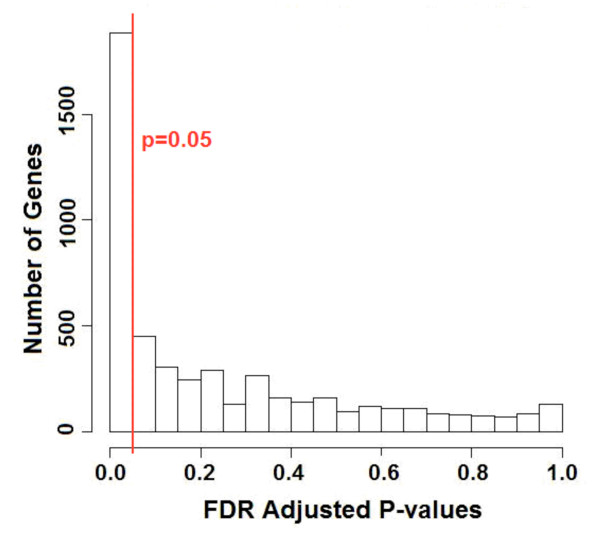
**Statistical significance of cross-experiment phase consistency**. Approximately 1,900 genes can be rejected at level 0.05 for the null hypothesis that their peak phases across experiments are distributed uniformly over a circular range.

Here, we are using all available experimental data, and using more information from these data than previously considered, so it is expected that there will be statistical evidence for a larger number of cell cycle oscillating genes. Fig. 5, which uses only phase information, suggests there could be between 1400 and 3200 cell cycle oscillating genes. We applied a test of circular uniformity across experiments, using only peak phase information, and this test suggested around 1,900 cell cycle oscillating genes (Fig. 6). Finally, to most efficiently use all available information, we did permutation testing using PCM, as follows: for each gene, every time course experiment was shuffled with respect to the time axis and with the median expression level of a random gene (i.e. *qE*_*gj *_= 0.5, see Methods). A "PCM" score was calculated based on the shuffled measurements in all experiments. This was repeated 1,000 times per gene to obtain the p-value based on its PCM scores from shuffled (i.e., randomized) data. After FDR adjustment of the p-values, the randomization procedure suggests that there are about 2,554 cell cycle oscillating genes. In other words, we estimate about 40-50% of all *S. pombe *genes to be cell cycle oscillating to some degree, however slight. Many of these new, weakly-oscillating genes peak in G2 phase, but many peak at other times, especially at M/G1, the same time as most strongly-oscillating genes (Additional file 2; Fig. S1).

**Figure 7:**
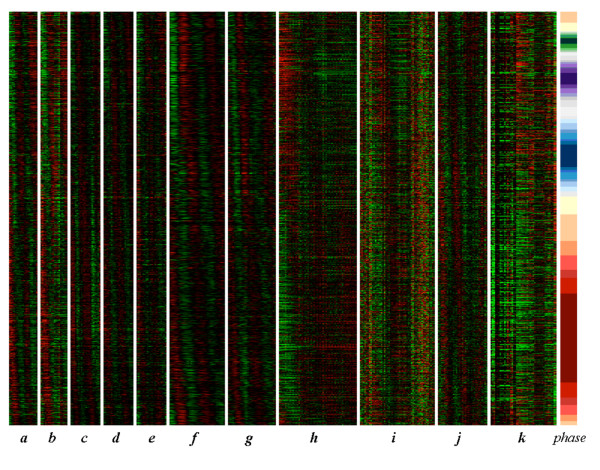
**Periodic oscillation of 1,275 "non-periodic" genes**. After removal of genes identified as periodic in the Oliva et al. and Marguerat et al. studies from the top 2,000 PCM ranked genes, there are 1,275 genes remaining. In this figure, these 1,275 genes are stacked top to bottom in phase order. Cyclic behavior is apparent. The time courses *a-e *are from Rustici et al., *f-g *from Peng et al., and *h-j *from Oliva et al. Block *k *consists of samples from transcription factor knockout and overexpression experiments (i.e., this block does not contain a cell cycle experiment). The color band "Phase" marks the phase distribution (red-G2, green-M, purple-G1, blue-S). A high resolution version is available in additional file 3.

For visual confirmation of the idea that there could be as many as 2,000 weakly-oscillating genes, we took the top 2,000 genes in the PCM-ranked list, and removed all the genes that were designated cell cycle oscillating by either Oliva et al. or Margureat et al., leaving about 1,275 genes (i.e., 1,275 genes hitherto considered "non-oscillating"). (Not all Oliva or Marguerat genes were in the top PCM 2000 genes, so the number of "non-oscillating" genes is more than 2000 minus the 750 genes of Oliva et al.) The expression patterns of these 1,275 genes are shown in Fig. 7. The genes are stacked on top of each other by time of peak expression. Fig. 7 clearly shows that to the eye, many or most of these genes appear to be cell cycle oscillating (high resolution version in Additional File 3). The statistical evidence for cell cycle oscillation of roughly 2,000 genes is strengthened by this visual evidence, even though the oscillation of the worst 1,000 of these genes is extremely weak. The significance, of any, of these very weak oscillations is addressed in the Discussion.

### An enhanced cluster of ribosomal biogenesis genes

**Figure 8:**
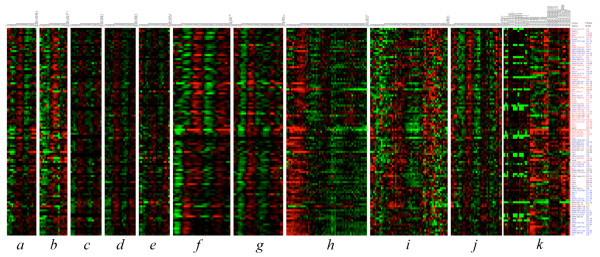
**The enhanced ribosomal biogenesis cluster *C*_*ribo*_**. The 103 genes constituting the "C_*ribo*_" cluster, expression of these genes peaks in G2 phase. The genes in this cluster are marked with '*C*_*ribo*_' in the PCM rank list (Additional file 1). The time courses *a-e *are from Rustici et al., *f-g *from Peng et al., and *h-j *from Oliva et al. Block *k *consists of samples from transcription factor knockout and overexpression experiments. A high resolution version is available in additional file 4.

Given the increased statistical power to identify weakly-oscillating cell cycle genes, we performed clustering with the top 2,000 genes from the PCM-based rank list. Since this pool of genes includes many weakly-oscillating genes, we used a novel two-step refinement strategy to allow stepwise filtering to produce biologically meaningful clusters. In each step an algorithm that is robust and well suited for that step was applied (see Methods). We obtained an enhanced ribosomal biogenesis cluster *C*_*ribo *_with 103 genes specific to early to mid-G2 phase (Fig. 8, high res version Additional file 4), which was a 137% increase with respect to the original ([9]) size of this cluster. Of the newly included genes, 52% had PCM ranks between 750 and 2000 (i.e. weakly oscillating) and many were annotated in GO as ribosomal proteins (e.g. *dbp10*, SPBC3F6.04C, SPBC365.04C, SPAC823.04, SPBC1604.06C, *fcf2*, *moe1*). Motif analysis of the intergenic regions upstream of these genes revealed a novel and statistically significant motif BYTCGTTA (where B is C or G or T, and Y is C or T) with p-value 3.7 × 10^-18^. The motif was found in 56% (58/103) of all the genes in *C*_*ribo *_as well as in 52% (47/91) of the known orthologs of *C*_*ribo *_genes in *S. japonicus*. This motif could be the binding site of a DNA binding protein that helps regulate the genes of this cluster.

## Discussion

Most microarray cell cycle studies in the past decade have modeled the periodic time courses with Fourier summation of sinusoidals [4-6,8,9,11]. However, in general such a Fourier score is independent of the time course's phase angle, and most microarray cell cycle analyses have not explored peak phase information in a systematic manner. While some approaches in the past have modeled a gene's phase for detection of periodic oscillations [24,25] or used it to identify the bottleneck genes [26], others have explicitly ignored it [27]. Although Marguerat et al. noted the phase variation [11], no study that we are aware of has used it for combining multiple experiments [6,9,11,28]. The main contribution of our Phase-Coupled Meta-analysis is the use of peak phase information within a novel measure - the PCM score - to combine ten genome-wide studies for identification of cell cycle oscillating genes in *S. pombe *with substantial increase in power and prediction accuracy.

The premise of our study is simple: consistency in the cell cycle phase of a gene's peak expression across multiple independent experiments is an indicator of genuine cell cycle oscillation. Using information about the consistency of the phase of peak expression, and adding it to information about the strength of oscillation, will give a better result than the strength of oscillation alone. By design, the PCM score shows more specificity and sensitivity for identifying genes with high phase consistency than, for instance, the previous meta-analysis by Marguerat et al. which combined p-values for periodicity and expression (Fig. 3); the relatively lower phase variance of genes that received higher ranks by PCM than by Marguerat et al. is statistically significant (p-value < 2.2 × 10^-16 ^for two-sample *t *test). Thus our PCM meta-analysis helps to discover genes that are weakly but consistently oscillating.

We observed low circular variance (< 0.4) across 10 experiments for more than 50% of all genes in *S. pombe *(Additional file 2; Fig. S2a). In fact, the count increased to more than 2,900 genes when the experiment in which a gene deviated most from its median phase was excluded from variance computation (Additional file 2; Fig. S2b). Under the assumption that a gene which is not cell cycle oscillating should peak, with whatever amplitude, at random phases in different independent cell cycle experiments, we tested every gene for phase uniformity over a circular range [29], and for 1,884 genes, as shown in Fig. 6, the uniform phase distribution hypothesis was rejected at significance level 0.05 (of which 1,189 genes were rejected at level 0.01). Indeed a closer look at the peak phases of genes that were not identified as periodic by earlier studies show significant phase consistency over several experiments (Fig. 4). These observations motivated us to design the PCM score for systematic phase-coupled meta-analysis of multiple time course experiments, and to re-rank all genes in *S. pombe *based on it.

The effectiveness of PCM scoring can be illustrated using the contrasting examples of SPAC6G9.06C and SPAC27D7.09C. The former (a.k.a. *pcp1*) is a weakly oscillating but known cell cycle gene; Fig. 2c shows PCM vectors of moderate magnitude representing its weak oscillation in ten experiments. Marguerat et al. did not rank the gene among the top 1,000 nor included it as cell cycle oscillating. However, we note that the same vectors show high peak phase consistency with similarity of direction. As a result, the gene obtained PCM rank of 259. Indeed, the GO functional annotation for the gene is as follows: chromosome segregation; microtubule nucleation; mitotic cell cycle spindle assembly checkpoint; mitotic metaphase/anaphase transition. Moreover, its *S. cerevisiae *ortholog YDR356W is known to be phosphorylated by Mps1p in cell cycle-dependent manner. On the other hand, SPAC27D7.09C is in general strongly expressed, as seen in the form of large magnitude PCM vectors in Fig. 2d. However it is a known heat shock stress responder which has mediocre cell cycle peak phase consistency across ten experiments as can be observed by the inconsistent direction of the vectors. Marguerat et al. ranked it among their top 300 genes and included it as cell cycle oscillating while PCM penalized its phase inconsistency and assigned it a much lower rank of 1,464.

Many of the new cell cycle oscillating genes peak in early to mid G2 phase, and trough in mitosis. A cluster of 44 similar genes was described by Oliva et al. [9] as the "kap123" cluster, which contains many genes involved in ribosome biogenesis (note that this cluster does not contain many actual components of the ribosome; it contains only a few actual ribosomal protein genes. The vast majority of the ribosomal protein genes form a separate cluster that we do not discuss here.). Using the new cell cycle genes, and a two-step clustering algorithm, we enhanced the "kap123" cluster to form the *C*_*ribo *_cluster of 103 genes, many of which pertain to ribosome biogenesis. The presence of a larger number of genes allowed us to identify a statistically significant motif {TCG}{TC}TCGTTA in more than half of all the genes in *C*_*ribo*_. The motif was found to be conserved in the regulatory sequences of a similar percentage of orthologs in fission yeast *S. japonicus*. Searching the TRANSFAC binding site database yields close matches with motifs for several Hox proteins: chicken HOXA4 (entry: TTCTCGTTATCT) and human HOX11 (TGACCGgTCGTTAA). Interestingly, HOX11 is a homeodomain transcription factor which is known to be cell-cycle regulated [30]. Further, it interacts directly with protein phosphatases that normally regulate cell cycle check point in G2-phase [31]. We note that in higher eukaryotes, ribosomal RNA transcription (which is dependent on RNA polymerase I) is inhibited during mitosis; this might also be true in *S. pombe*, and if so, the matching trough in the ribosomal biogenesis genes we see in *C*_*ribo *_might be a response to a lack of ribosomal RNA.

Our work here carries on from the analysis of Marguerat et al. [11], in part because we wished to show the power of our new method by comparing our results to previous results for the same data. Thus, we have used the same data as Marguerat et al., and we have also used their "regulation" and "periodicity" scores to calculate the magnitude of our vectors. However, Marguerat et al. concluded that the data supported identification of about 500 cell cycle oscillating genes, while we believe it supports identification of about 2000 such genes. There are at least two reasons for this difference. First, because we are using consistency of peak phase in addition to regulation scores and periodicity scores, we are using more of the information inherent in the data, not just within-experiment but also across experiments, and this gives us greater power. Second, we make different assumptions than Marguerat et al. with regard to permutation testing. We use the null hypothesis that all variations in the experimental measurements in a time course are due to random noise; our p-values are relative to this null hypothesis. In contrast, Marguerat et al believe that random noise null hypothesis may be naive; for instance, there could be effects such that experimental measurements in adjacent time points are correlated and thus not independent. If so, p-values calculated by permutation testing on the null hypothesis of completely random noise would be too small [32]. Marguerat et al. made an ad hoc adjustment for this possibility and then normalized (and raised) all their initially calculated p-value distributions around the median p-value of each distribution. We feel that this is a somewhat aggressive adjustment that increases p-values significantly, thus significantly decreasing the apparent number of cell-cycle oscillating genes. While we completely agree with Marguerat et al., and Futschik and Herzel [32], that the null hypothesis of completely random noise may be too simple, which if true would lead to p-values that are too small, on the other hand, there is no objective, quantifiable alternative, and we do not wish to make an ad-hoc adjustment. Thus, at least for the time being, we feel that the null hypothesis of random noise is the best we can do. In any case, regardless of statistical arguments, it is visually and intuitively clear from Fig. 7 that there are many more than 500 cell cycle oscillating genes.

Marguerat et al. also argue for a relatively small number of cell cycle oscillating genes using benchmark sets of known cell cycle oscillating genes. They choose three sets of benchmark cell cycle genes, consisting of 40 very strongly oscillating genes whose periodicity has been demonstrated in small scale experiments (set B1); genes whose promoters are bound by the known cell cycle transcription factors Cdc10, Res1, Res2 (all three of these being components of MBF) or Fkh2 (set B2); and genes shown in expression experiments to be the targets of the transcription factors Ace2, Sep1, or Cdc10 (set B3). Marguerat et al. find that their top-ranked 500 cell cycle genes are enriched for the genes in these benchmark sets, but that there is little if any enrichment after the top 500. However, we feel this argument is unconvincing. The benchmark sets contain strongly oscillating cell cycle genes, or genes controlled by a small number of powerful cell cycle transcription factors. Cell cycle oscillating genes of distinctly different kinds (e.g., genes weakly regulated by other transcription factors, or by RNA half-life, or by chromatin condensation) are not in any of the benchmark sets at all. Thus, for example, many of the genes we find ranked between 500 and 2000 are in the *C*_*ribo *_cluster described above. These are not strongly oscillating, and are not regulated by any of the transcription factors used for benchmark sets 2 or 3, and are not significantly (if at all) represented in any of the benchmark sets. In short, since the benchmark sets consist of strongly oscillating genes controlled by a few known transcription factors, it is not surprising that these genes are not enriched amongst weakly oscillating genes controlled in other ways.

### The biological relevance of 2000 cell cycle oscillating genes

By "cell cycle oscillating" gene, we mean a gene whose expression oscillates up and down, however slightly, as a function of position in the cell cycle. Traditionally, one thinks in terms of genes whose cell cycle oscillation is adaptive; that is, genes where there is a selective advantage to the organism if gene expression oscillates. Examples of such genes are *cdc22*, encoding ribonucleotide reductase, and the histone genes, encoding histone proteins. Ribonucleotide reductase is needed only for making deoxyribonucleotides in preparation for immediate DNA synthesis; otherwise it is a metabolically-expensive hindrance. Similarly, histone proteins are needed only for making nucleosomes during DNA replication; otherwise they interfere with proper DNA metabolism. We refer to such genes, where the oscillation is adaptive and purposefully controlled by the cell, as "cell cycle regulated" genes.

Recently, the issue of "how many genes are cell cycle regulated" has received some attention [33,34]. Although we believe that 2000 fission yeast genes, or even more, do oscillate in expression at least slightly as a function of cell cycle, the oscillations of many of these genes may not be adaptive. Instead, many oscillations may be indirect, unavoidable consequences of the cell cycle, and may have no adaptive significance. For instance, in preparation for mitosis, chromosomes condense. It seems likely that chromosome condensation might interfere with transcription. Indeed, in higher eukaryotes, bulk transcription is greatly inhibited during mitosis. Genes whose transcription is inhibited during mitosis, perhaps as either a direct or indirect effect of chromosome condensation, would be detected in microarray experiments as cell cycle oscillating genes.

Thus, we suggest that there may be two classes of cell cycle oscillating genes. First, there are what we call "adaptive" oscillations, i.e., oscillations that are a selective advantage for the organism; *cdc22 *and histones are examples of genes with adaptive oscillations. Second, there might be what we call them "incidental" oscillations, i.e., oscillations that are not adaptive, but instead are a consequence of some other cell cycle event, such as chromosome condensation. At present there is no good way of distinguishing adaptive cell cycle regulation from incidental oscillation, but it seems likely that most strong oscillations, associated with a cell cycle transcription factor, are probably adaptive, while weak oscillations, not associated with a cell cycle transcription factor, may (or may not) be incidental.

Finally, we note that while evidence has accumulated for more and more cell cycle oscillating genes, parallel results have been obtained for genes oscillating with a circadian rhythm [35-37]. Exactly as in the case of cell cycle genes, microarrays have allowed the accumulation of large amounts of data for circadian oscillations, and exactly as in the case of cell cycle, increasingly powerful statistical methods have allowed the discovery of an increasingly large number of genes with a circadian oscillation. The most recent studies suggest that nearly all mammalian genes have at least a slight circadian oscillation [37]. As in the case of the cell cycle genes, these circadian rhythms may sometimes be adaptive, and sometimes indirect and incidental.

## Conclusion

In conclusion, we have developed a new meta-analysis method that uses more of the information inherent in microarray studies of cell cycle gene expression. This extra information allows us to detect a larger number of cell cycle oscillating genes. Because the proportion of oscillating genes is very large - one quarter to one half of all genes in the organism - we suggest that many of them may be oscillating for incidental reasons, rather than because the oscillation is necessarily adaptive.

## Authors' contributions

SP designed research, performed PCM analysis and wrote the manuscript; RG performed the motif analysis; CSK performed the clustering analysis; BF designed research and wrote the manuscript. All authors read and approved the final manuscript.

## Supplementary Material

Additional file 1 **PCM rank list of fission yeast genes**. The spreadsheet provides the genome-wide rank of fission yeast genes due to Phase-Coupled Meta-analysis (PCM). It also shows whether a gene was reported by Oliva et al. or Marguerat et al. as periodic, its median phase over all experiments and its circular variance, and to which cluster due to Oliva et al. it belongs. The last field also indicates membership in the new *C*_*ribo *_cluster. Click here for file

Additional file 2 **Supplementary figures**. Fig. S1 plots the distribution of cell cycle phases for genes from the PCM rank-list. Fig. S2 plots the genome-wide distribution of the circular variance of every gene's phases across experiments. Click here for file

Additional file 3 **High resolution plot**. High resolution version of Fig. 7. Click here for file

Additional file 4 **High resolution plot**. High resolution version of Fig. 8. Click here for file
