# Supplementary material for: Phase Coupled Meta-analysis: sensitive detection of oscillations in cell cycle gene expression, as applied to fission yeast
Source: BMC Genomics. 2009 Sep 17;10:440. doi: 10.1186/1471-2164-10-440 (PMC2753555; doi:10.1186/1471-2164-10-440)
Supplement: Additional file 2 — Supplementary figures. Fig. S1 plots the distribution of cell cycle phases for genes from the PCM rank-list. Fig. S2 plots the genome-wide distribution of the circular variance of every gene's phases across experiments. [file 1471-2164-10-440-S2.PDF]

Supplementary figures

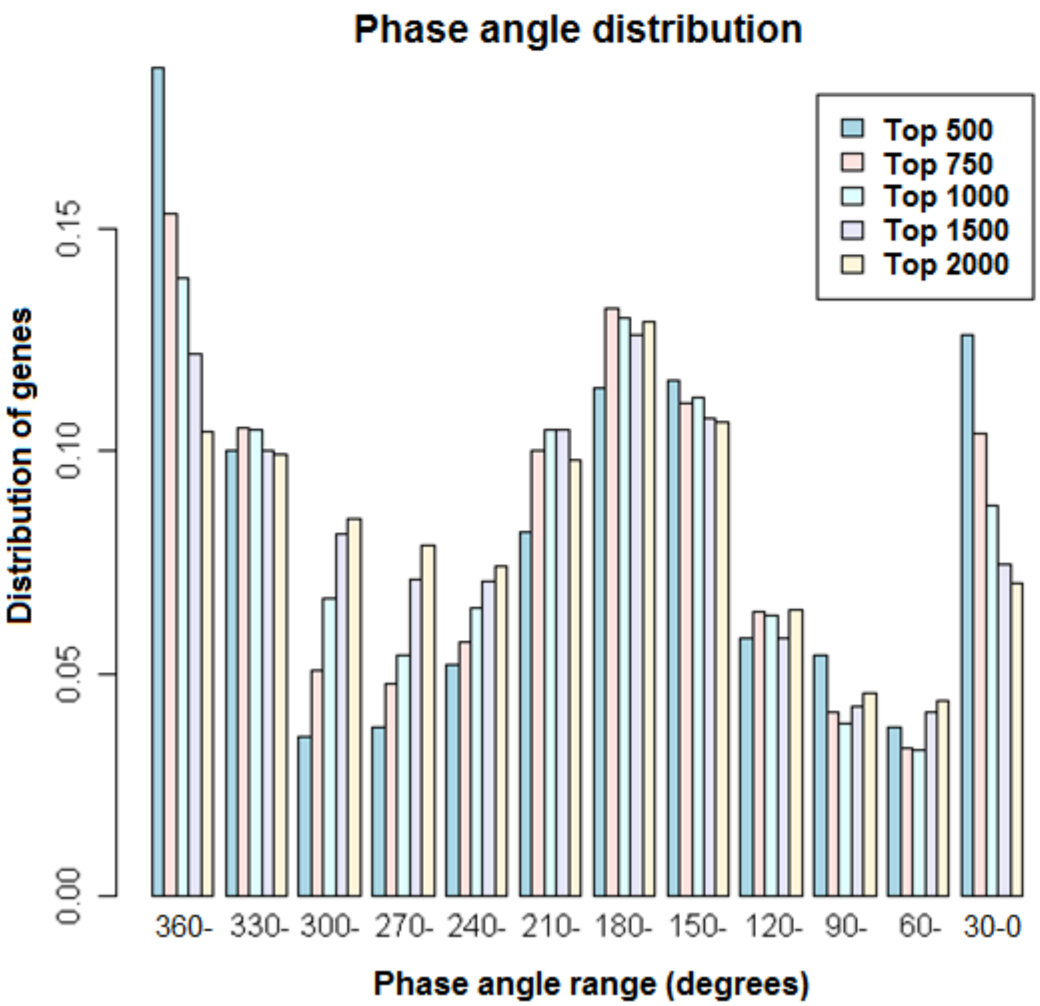

Fig. S1. Phase angle distribution. The distributions for the top 500, 750, 1000, 1500 and 2000 genes from the PCM rank list are shown. The cell cycle phases are roughly distributed as G1:0-55 degrees, S:55-110 degrees, G2:110-325 degrees, and M:325-360 degrees.

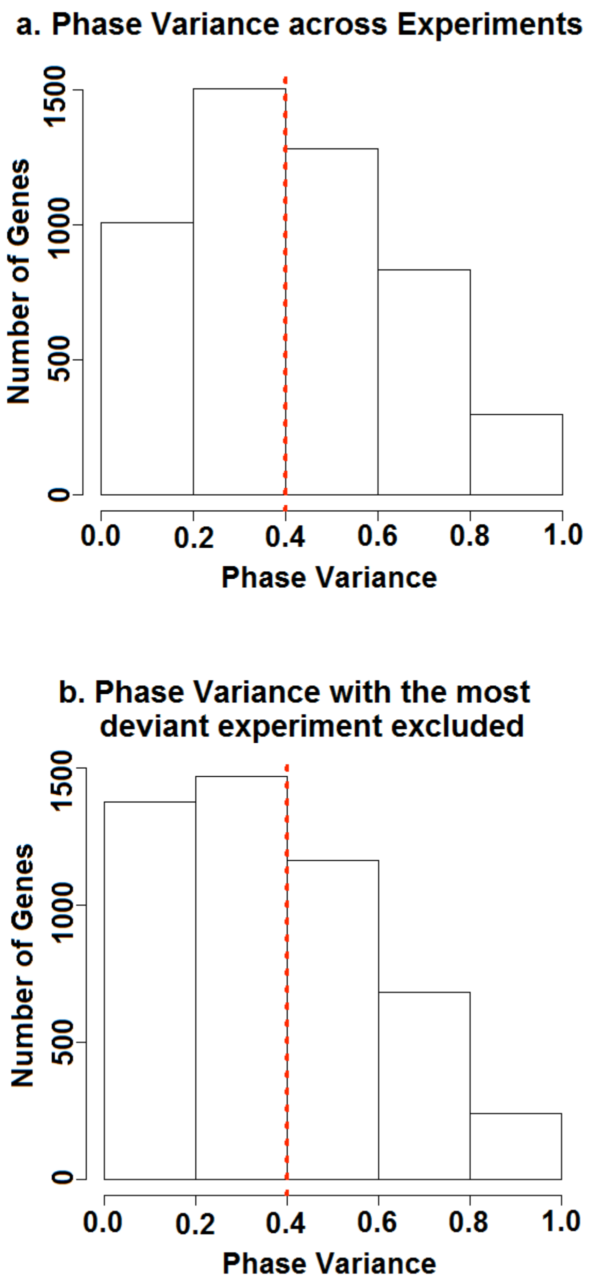

Fig. S2. Genome-wide distribution of cross-experiment phase consistency. Circular variance of the peak phases of each gene in all experiments is computed as a measure of its phase consistency, and the distribution of variances over all genes is shown. The dotted red lines separate the genes with circular variance less than or equal to 0.4. In plot (a), all available experiments for every gene were used in the computation of circular variance. Plot (b) is similar except the experiment with the phase that is most deviant from a gene's median phase was excluded from the computation of circular variance.
