# Supplementary figures and images for: Phase Coupled Meta-analysis: sensitive detection of oscillations in cell cycle gene expression, as applied to fission yeast
Source: BMC Genomics. 2009 Sep 17;10:440. doi: 10.1186/1471-2164-10-440 (PMC2753555; doi:10.1186/1471-2164-10-440)

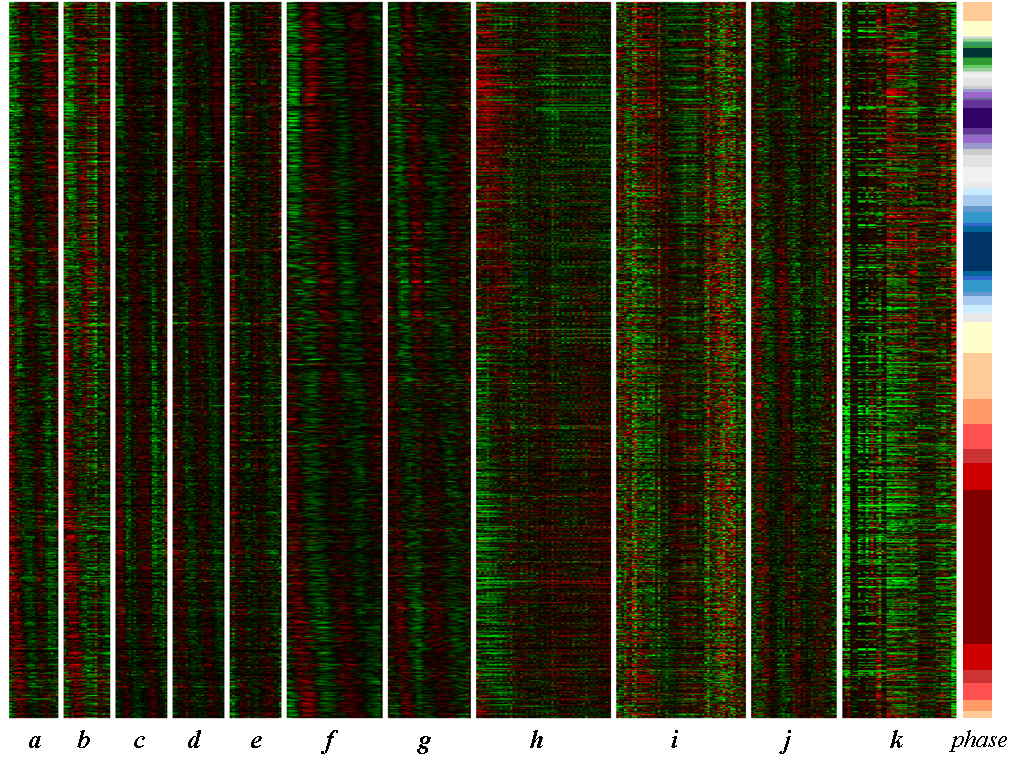

Supplement: Additional file 3 — High resolution plot. High resolution version of Fig. 7. [file 1471-2164-10-440-S3.TIFF]

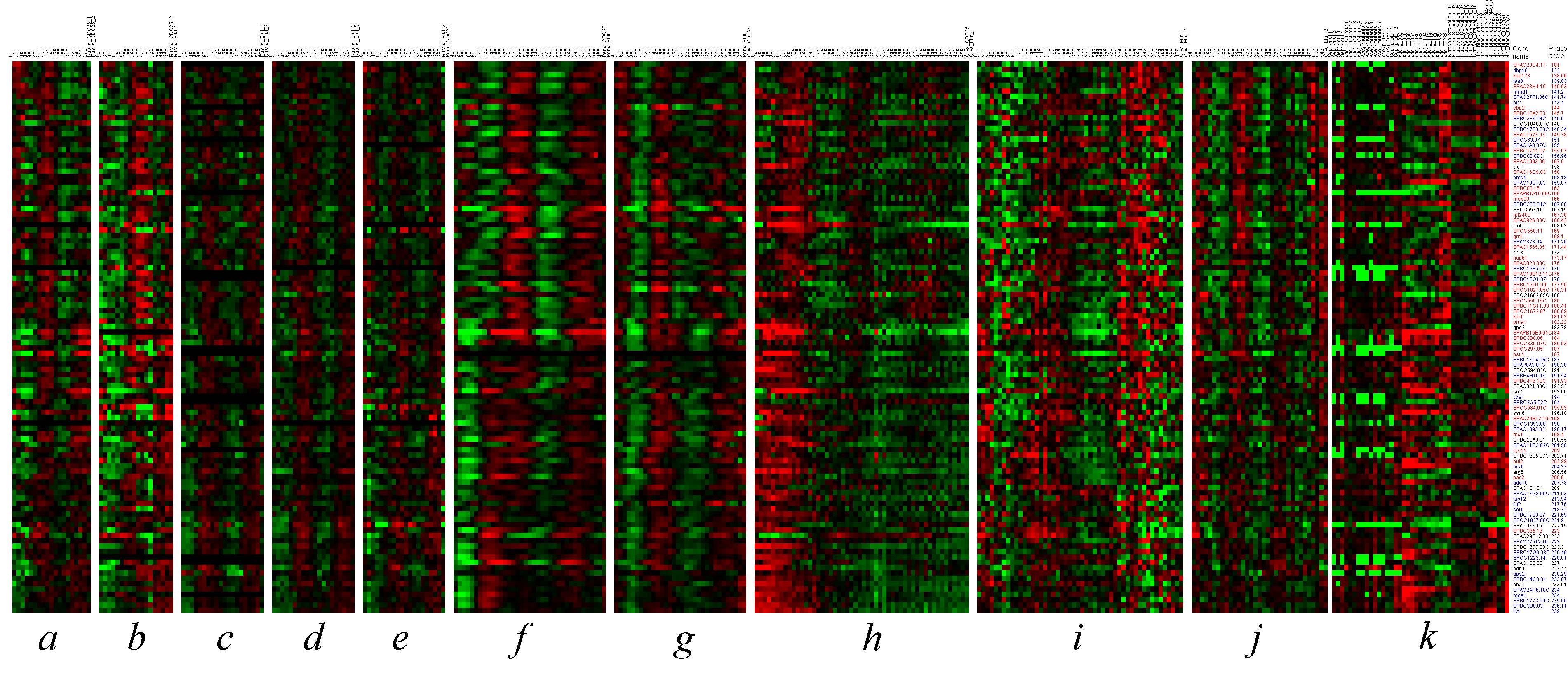

Supplement: Additional file 4 — High resolution plot. High resolution version of Fig. 8. [file 1471-2164-10-440-S4.TIFF]
